# PM_2.5_ air pollution contributes to the burden of frailty

**DOI:** 10.1038/s41598-020-71408-w

**Published:** 2020-09-02

**Authors:** Wei-Ju Lee, Ching-Yi Liu, Li-Ning Peng, Chi-Hung Lin, Hui-Ping Lin, Liang-Kung Chen

**Affiliations:** 1 Aging and Health Research Center, National Yang Ming University, Taipei, Taiwan; 2 Department of Family Medicine, Taipei Veterans General Hospital Yuanshan Branch, Yilan County, Taiwan; 3 Institute of Hospital and Health Care Administration, National Yang Ming University, Taipei, Taiwan; 4 Center for Geriatrics and Gerontology, Taipei Veterans General Hospital, No. 201, Sec. 2, Shih-Pai Road, Taipei, 11217 Taiwan; 5 Department of Health, New Taipei City Government, New Taipei, Taiwan

**Keywords:** Medical research, Risk factors, Signs and symptoms

## Abstract

Frailty is common among older people and results in adverse health outcomes. We investigated whether exposure to PM_2.5_ is associated with frailty. This cross-sectional study involved 20,606 community-dwelling participants aged ≥ 65 years, residing in New Taipei City, Taiwan. Analytic data included phenotypic frailty, disease burden by Charlson Comorbidity Index (CCI), urban or rural residence, and household income. PM_2.5_ exposure was calculated from air quality monitoring records, with low exposure defined as the lowest quartile of the study population. 1,080 frail participants (5.2%) were older, predominantly female, had more comorbidities, lived rurally, and had low PM_2.5_ exposure (all *p* < 0.001). In multinomial logistic regression analyses, the likelihood of high PM_2.5_ exposure was higher in prefrail (OR 1.4, 95% CI 1.3–1.5) and frail adults (OR 1.5, 95% CI 1.2–1.9) than in robust individuals, with stronger associations in those who were male (frail: OR 2.1, 95% CI 1.5–3.1; prefrail: OR 2.2, 95% CI 1.9–2.6), ≥ 75 years old (frail: OR 1.8, 95% CI 1.3–2.4; prefrail: OR 1.5, 95% CI 1.3–1.8), non-smokers (frail: OR 1.6, 95% CI 1.3–2.0; prefrail: OR 1.4, 95% CI 1.2–1.5), had CCI ≥ 2 (frail: OR 5.1, 95% CI 2.1–12.6; prefrail: OR 2.1, 95% CI 1.2–3.8), and with low household income (frail: OR 4.0, 95% CI 2.8–5.8; prefrail: OR 2.7, 95% CI 2.2–3.3). This study revealed a significant association between PM_2.5_ exposure and frailty, with a stronger effect in vulnerable groups.

## Introduction

Together with population aging, frailty has garnered considerable interest among clinical researchers and public health professionals, because its reversible and dynamic nature offer promise for disability prevention. Frailty is a geriatric syndrome characterized by declining physiological reserves and increasing vulnerability, in which disrupted homeostasis due to accumulated inter-systemic deficits results in disproportionate health consequences in response to minor stressors^1^. This complex age-related pathophysiology is considerably influenced by combined environmental and genetic factors^2^.

Air pollution is the foremost reversible environmental factor associated with premature death or disability^3^. Accruing epidemiological evidence supports associations between exposure to air pollution and adverse outcomes such as cardiovascular events^4–6^, cancer^7^, Alzheimer’s disease^8^ and mortality^9^. Fine particulate matter < 2.5 μm in diameter (PM_2.5_) is of greatest health concern, not only such small particles can penetrate deeper into the lungs than larger ones do, but also they are likely to be made up of a more toxic mix^4^.

Possible biological mechanisms explaining the health consequences of PM_2.5_ exposure, include oxidative stress, endothelial dysfunction, and pro-inflammatory effects such as elevated interleukin 6 levels^4,10^; frailty shares similar pathoetiology^11,12^. Previous studies have investigated potential associations between fine airborne particulates and frailty^13–15^. A study of 2059 non-smokers from the United States National Health and Nutrition Examination Survey found a positive association between secondhand tobacco smoke and frailty^13^. Others confirmed an association between PM_2.5_ and frailty, measured using a frailty index, among post-myocardial infarction patients who were inherently vulnerable to frailty^14,15^. However, it remains unknown whether or not PM_2.5_ exposure is associated with frailty in the general population. Hence, we investigated associations between annual PM_2.5_ exposure and frailty among community-dwelling older people, and in potentially vulnerable subgroups.

## Results

The prevalence of frailty in this study sample was 5.2%; compared to the robust people, 1,080 frail participants were significantly older, predominantly female, had higher disease burden, lived rurally, and had lower annual PM_2.5_ exposure (Table 1). Adjusted for age, sex, smoking, CCI, and urbanicity, frail and pre-frail statuses were significantly associated with high annual PM_2.5_ exposure. Compared to robust older adults, Odds ratio (OR) for those with prefrail and frailty were 1.4 (95% confidence interval (CI) 1.3–1.5) and 1.5 (95% CI 1.2–0.9). Weakness (OR 1.4, 95%CI 1.3–1.6) and low activity (OR 1.6, 95%CI 1.4–1.7) were associated with high PM_2.5_ exposure, but other phenotypic components of frailty were not (Table 2).

**Table 1:** Demographic and health-related characteristics by frailty status.

Data show number (%); mean ± SD; median (interquartile range)	Entire cohort	Robust	Prefrail	Frail	*p* value	*p* for trend
Number (%)	20,606 (100.0)	10,384 (50.4)	9,142 (44.4)	1,080 (5.2)		
Age (years)	72.9 ± 6.6	71.5 ± 5.6	73.8 ± 7.0	78.0 ± 7.8	** < 0.001**	** < 0.001**
Age ≥ 75 years	7,231 (35.1)	2,770 (26.7)	3,746 (41.0)	715 (66.2)	** < 0.001**	** < 0.001**
Male	9,496 (46.1)	4,990 (48.1)	4,020 (44.0)	486 (45.0)	** < 0.001**	** < 0.001**
Charlson Comorbidity Index ≥ 2	887 (4.3)	374 (3.6)	413 (4.5)	100 (9.3)	** < 0.001**	** < 0.001**
Smoker	1,262 (6.1)	619 (3.0)	575 (2.8)	68 (0.3)	0.615	0.357
Urbanization index ≥ 4	3,306 (16.0)	1,151 (11.1)	1822 (19.9)	333 (30.8)	** < 0.001**	** < 0.001**
PM_2.5_ (μg/m^3^)	17.7 (16.3, 18.8)	17.7 (16.3, 18.8)	17.7 (16.3, 18.0)	16.8 (14.7, 18.0)	** < 0.001**	** < 0.001**
Household income < 19,677 USD^a^	10,251 (49.8)	4,607 (44.4)	5,026 (55.0)	618 (57.2)	** < 0.001**	** < 0.001**

*SD* standard deviation, *USD* United States Dollars.

^a^Converted from New Taiwan Dollars (NTD) at a rate of 1 USD = 31 NTD.

Bold type denotes statistical significance.

**Table 2:** Association between frailty status and individual phenotypes, and PM_2.5_ in logistic regression analyses.

	High PM_2.5_	High PM_2.5_		Log PM_2.5_	
	number/total	Odds ratio (95% CI)^a^	*p* value	Odds ratio (95% CI)^a^	*p* value
**Frailty status**					
Robust	7,290/10,384	1 (reference)		1 (reference)	
Prefrail	5,950/9,142	1.4 (1.3–1.5)	** < 0.001**	1.7 (1.2–2.6)	** < 0.001**
Frail	605/1,080	1.5 (1.2–1.9)	** < 0.001**	1.1 (0.5–2.4)	0.738
**Frailty phenotype**					
Weakness	3,362/5,220	1.4 (1.3–1.6)	** < 0.001**	1.7 (1.1–2.9)	**0.003**
Slowness	1637/3,091	1.0 (0.8–1.1)	0.507	1.2 (0.7–1.9)	0.462
Weight loss	219/349	0.8 (0.6–1.2)	0.261	1.5 (0.4–5.8)	0.599
Exhaustion	608/970	0.9 (0.7–1.2)	0.466	0.3 (0.1–0.7)	**0.005**
Low activity	3,668/5,586	1.6 (1.4–1.7)	** < 0.001**	1.3 (0.8–2.1)	0.266

*CI* confidence interval.

^a^Multinomial logistic regression adjusted for age, sex, smoking, Charlson Comorbidity Index, and urbanization.

Bold type denotes statistical significance.

**Figure 1:**
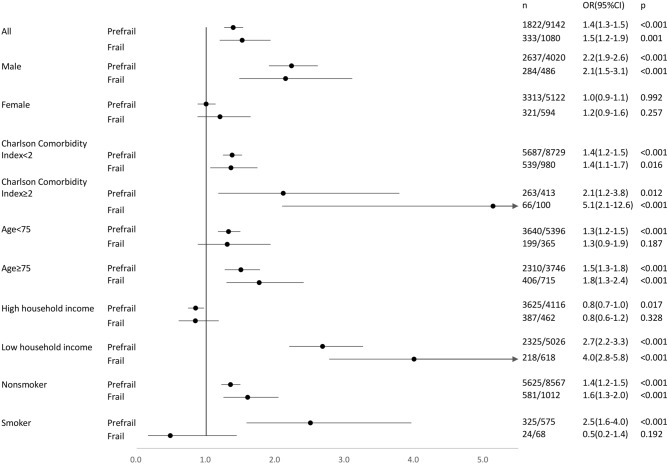
Association between high PM_2.5_ exposure and frailty status by age, sex, smoking, disease burden, and household income.

Figure 1 and Supplementary Table S1 in the supplement summarize the results of multivariate multinomial logistic analysis of associations between frailty and PM_2.5_ across various subgroups. The likelihood of people with high PM_2.5_ exposure being frail was generally higher among vulnerable groups such as those who were older, had higher disease burden, or with low household income. Since the lowest PM_2.5_ exposure level in rural areas was zero, we used subsamples from urban areas to test associations between frailty and PM_2.5_, with results similar to those for the entire sample.

Frailty reflects aging and the progression of disease, including comorbid chronic conditions. The association between frailty and PM_2.5_ seems to be driven by weakness and low activity—which presumably were also associated with chronic disease (respiratory disease, cardiovascular disease, cancer, etc.) and were also likely associated with PM_2.5_. Hence, we conducted a sensitive analysis that adjusted for chronic diseases of hypertension, diabetes, heart disease, stroke and chronic kidney disease. Results were similar (Table supplement S2).

## Discussion

This study discovered a significant association between frailty and fine airborne particulates in a large population of community-dwelling elders. The associations were stronger among men, people older than 75 years, with higher disease burden, non-smokers, and those with lower household income.

Most studies on air pollution have focused on conventional disease-oriented outcomes, such as cardiometabolic disease, cancer, or mortality^5,7,9^, rather than on functional ones; few studied frailty outcomes. However, frailty per se is emerging as a prime candidate for disability prevention in the modern era of function-centered medicine. The role of frailty in modifying pulmonary function deterioration^16^ and post-myocardial infarction mortality^15^ has been reported. Ten-year follow-up of 848 non-frail myocardial infarction patients showed increased likelihood of becoming frail^14^. Hong Kong investigators found differences in frailty prevalence between administrative districts but failed to prove an association with air pollution, due to limited exposure data^17^. Our findings affirm an association between PM_2.5_ and frailty, and extend this from heart attack survivors to the general population.

Phenotypic manifestations of frailty are better suited to investigating pathological mechanisms^1^ than are operational definitions based on a frailty index^18^. As far as we know, this is the first evidence to of an association between PM_2.5_ exposure and phenotypically-defined frailty in the general population. Among five established frailty phenotypes, weakness was significantly associated with PM_2.5_ exposure levels. Weakness, which is an essential component of mobility type frailty and sarcopenia^19,20^, increased accuracy in predicting cardiovascular mortality in previous studies^21^. Given the strong association between handgrip strength and mortality, weakness may be an intermediate state on the way to eventual mortality due to air pollution.

Frailty, disease burden, and socioeconomic status have synergistic effects on disability^22,23^. We found the likelihood of people with high PM_2.5_ exposure levels being frail to be around four–five-fold higher among vulnerable groups, such as those with lower household income and higher disease burden, consistent with previous studies^14,15^. Likewise, the association was stronger in people aged ≥ 75 years compared to younger ones, corroborating other evidence that PM_2.5_ exposure has a greater impact in older people^15^. Taken together, the results of this study suggest that a stronger association between PM_2.5_ exposure and frailty in vulnerable groups may be particularly inimical to health outcomes.

Although frailty is more prevalent in women, the association between frailty and PM_2.5_ exposure was only evident in men, even after adjusting for smoking. A time-series study in Paris found that men, but not women, had significantly increased risk of hospitalization for respiratory complaints associated with air pollution^24^; this was consistent with the sex difference we observed, but the mechanism is unknown and warrants further investigation.

Non-smokers with high PM_2.5_ exposure in our study population were more likely than smokers to be frail. This may reflect an unmeasured residual competing risk of active smoking that masked the health hazard of PM_2.5_. Misclassification bias might be another plausibility. Similarly, the European Study of Cohorts for Air Pollution Effects found a stronger association between air pollution and stroke in non-smokers than in smokers^6^.

Putative mechanisms explaining the health consequences of PM_2.5_ exposure include oxidative stress, inflammatory responses, and gene/epigenetic modifications—factors also implicated in aging and frailty pathogenesis^1^. Airborne pollutants may disrupt homeostasis and accelerate age-related declines in functional performance and capacity at cellular, organ and system levels^25^; multi-systemic homeostatic disruption would conduce to frailty and hinder successful aging. Previous studies that investigated the relationship between PM_2.5_ exposure and frailty, used an operational definition based on a frailty index which measures cumulative deficits^18^ rather than making direct phenotypic assessments^11^. Our study affirmed an association with directly-measured phenotypic frailty.

Although the mean annual PM_2.5_ level in New Taipei city was much lower than in many other parts of Asia, which routinely exceed 35 μg/m^3^,^4^ it is considerably higher than the optimal target of < 10 μg/m^3^ set by World Health Organization air quality guidelines^26^ and routine values of < 12 μg/m^3^ in the United States and Canada^4^. Findings from a Hong Kong study of 3,240 community-dwelling adults ≥ 65 years old, suggests that green neighborhood space may mitigate exposure to air pollution and reverse frailty^27^, which could be the basis of a national level strategy. Personal level monitoring may contribute to increased awareness and protection^4^.

This study had limitations. First, the cross-sectional design precluded exploration of a reverse causality between frailty and PM_2.5_ exposure. Second, air pollution data were obtained from ambient air monitoring stations rather than personal exposure levels, which prevented distinguishing between indoor and outdoor pollution. Third, urbanicity was determined according to an urbanization index developed a decade ago, which may not accurately represent shifted demographic characteristics.

## Conclusion

Our study detected a significant association between PM_2.5_ exposure and frailty in among the general population, which was stronger in vulnerable groups. These results are consistent with the hypothesis that exposure to PM_2.5_ disrupts multi-systemic homeostasis, suggesting that frailty prevention and intervention strategies should incorporate reducing air pollution.

## Methods

### Data sources and participants

Study data were excerpted from the New Taipei City Elderly Health Examination Database (NTCHD) and the Taiwan Air Quality-Monitoring Database (TAQMD). The NTCHD was established for early detection of physical conditions and promote senior health. All older adults ≥ 65 years old residing in New Taipei City could receive voluntary government-funded annual examinations face-to-face by physicians, and these examinations include anthropometry, health-related behaviors, physical and mental performance and biochemistry results.

Upon enrollment, participants gave written informed consent authorizing the New Taipei City Government to process health examination data for research and policy purposes. New Taipei City Department of Health removed all potentially identifying information to protect privacy and generate the anonymized NTCHD. Full details of recruitment and data collections procedures are described elsewhere^22^.

The present study included 26,026 potentially eligible NTCHD participants in 2016. Having excluded 5,420 with incomplete data, the residential areas of the final analytic sample of 20,606 were linked to the locations of TAQMD ambient air-quality monitoring stations to estimate daily PM_2.5_ exposures.

This study was designed and conducted in accordance with the principles of the Declaration of Helsinki. New Taipei City Department of Health approved the use of this anonymized dataset for research purposes, and waived the requirement for Institutional Review Board approval. The design and reporting format follow Strengthening the Reporting of Observational Studies in Epidemiology (STROBE) guidelines.

### Frailty

The Cardiovascular Health Study frailty criteria comprise unintentional weight loss, exhaustion, weakness, slowness, and low activity; people with three or more of these frailty phenotypes are classed as frail, those with one or two as prefrail, and those with none as robust^11^. Unintentional weight loss is defined as losing > 5% of body weight over the previous 12 months. Exhaustion was determined by affirmation of two questions from the Center for Epidemiologic Studies Depression Scale questionnaire^28^—‘‘I felt everything I did was an effort’’ and ‘‘I could not get going’’—on ≥ 3 days per week. Physical activity was calculated from the International Physical Activity Questionnaire short-form score, based on self-reported exercise and leisure time physical activities, and expressed as weekly energy expenditure^29^. Energy expenditure was calculated as the metabolic equivalent relevant to designated type of exercise times body weight and days of exercise. In this study, energy expenditure below 383 kcal/week in men or 270 kcal/week in women was defined as low physical activity according to Cardiovascular Health Study^11^. Walking speed was measured by a six-metre walk test at a normal pace from a moving start without deceleration. Weakness was defined as maximum dominant handgrip strength of < 26 kg for men or < 18 kg for women, and slowness as 6-m walk speed < 0.8 m/s^30^.

### Urbanization

An urbanization index developed by the Taiwan National Health Research Institute was used to determine the urbanization level of each participant’s residential area. This index, which was based on national census data including population density and aging, education levels, medical resources, and agricultural employment, classified 359 regions throughout Taiwan into seven strata from most urbanized (level 1) to the least (level 7)^31^. Urbanicity was dichotomized as urban (level ≤ 3) or rural (level ≥ 4), based on a previous study^32^.

### Other variables

Smoking was defined as using tobacco during the previous 6 months. Charlson Comorbidity Index (CCI) ≥ 2 indicated severe disease burden^33^. Household income data from the Taiwan Ministry of Finance was used as a proxy for socioeconomic status; median annual income equivalent to less than the population median of 19,677 United States Dollars (USD) was considered low.

### PM_2.5_ exposure

Daily PM_2.5_ level was excerpted from TAQMD. Average annual fine particulate matter concentration was calculated by accumulating daily PM_2.5_ levels from the 12 months prior to the index interview date; exposure in the lowest quartile (< 16.3 μg/m^3^) was classed as low.

### Statistical analysis

All analyses were performed using SAS statistics software, Version 9.4 for Windows (SAS Institute, Inc., Cary, NC, USA). A two-sided p-value of < 0.05 or 95% confidence interval (CI) that did not include the null hypothesis value were considered statistically significant.

The Kolmogorov–Smirnov D test was used to check whether numerical variables were normally distributed. Continuous variables with normal distributions were expressed as means plus/minus standard deviation, and those with non-normal distributions as median (first quartile, third quartile). Categorical variables were expressed as frequency/proportions. One-way ANOVA, Kruskal–Wallis Test, chi-square analysis, or Fisher exact test were used as appropriate to compare descriptive characteristics. Cochran-Armitage trend test was used to test for trends. PM_2.5_ concentrations were transformed logarithmically to approximately linearity assumption for logistic regression. Urbanicity exhibited collinearity with household income, hence income was not included in the statistical model. Univariable and multivariable multinomial logistic regression analyses, adjusted for age, sex, smoking, CCI, and urbanicity, were used to explore associations between frailty status and PM_2.5_. Multivariable multinomial logistic analysis employed statistical analysis on association between frailty and PM_2.5_. Frailty as a categorical variable severed as a dependent variable; PM_2.5_ and other confounders referred as independent variable. The statistical results showed ORs and CI comparing prefrail vs. robust and frail vs. robust, respectively. Log PM_2.5_ was input to the model after categorizing low/high PM_2.5_ for sensitivity analysis. Prespecified subgroup analyses included age (< 75 vs. ≥ 75 years), sex (male vs. female), disease burden (CCI < 2 vs. ≥ 2), smoking (no vs. yes), household income (≥ 19,677 USD vs. < 19,677 USD) and urbanicity (urbanization index ≤ 3 vs. ≥ 4).

## Supplementary information

Supplementary Information.

## Data Availability

The data sets used in this study cannot be shared at the current time due to data confidentiality agreements and sharing restrictions from data sources.
