# Supplementary material for: PM2.5 air pollution contributes to the burden of frailty
Source: Sci Rep. 2020 Sep 2;10:14478. doi: 10.1038/s41598-020-71408-w (PMC7468121; doi:10.1038/s41598-020-71408-w)
Supplement: Supplementary file 1 — Supplementary Information. [file 41598_2020_71408_MOESM1_ESM.docx]

**PM_2.5_ air pollution contributes to the burden of frailty**

**Short run title**: PM_2.5_ and frailty

Wei-Ju Lee MD,PhD^1,2^, Ching-Yi Liu RN,BS^2,3^, Li-Ning Peng MD,PhD^1.4^, Chi-Hung Lin MD,PhD^5^, Hui-Ping Lin PhD^5^, Liang-Kung Chen MD,PhD^1.4,^*

^1^Aging and Health Research Center, National Yang Ming University, Taipei, Taiwan

^2^Department of Family Medicine, Taipei Veterans General Hospital Yuanshan Branch, Yilan County, Taiwan

^3^Institute of Hospital and Health Care Administration, National Yang Ming University, Taipei, Taiwan

^4^Center for Geriatrics and Gerontology, Taipei Veterans General Hospital, Taipei, Taiwan

^5^Department of Health, New Taipei City Government, New Taipei, Taiwan.

***Correspondence to:** Liang-Kung Chen, Center for Geriatrics and Gerontology, Taipei Veterans General Hospital, No. 201, Sec. 2, Shih-Pai Road, Taipei 11217, Taiwan.

E-mail: [lkchen2@vghtpe.gov.tw](mailto:lkchen2@vghtpe.gov.tw)

**Supplemental table**

| **Supplementary Table S1** Multivariable multinomial logistic analysis of associations between frailty and PM_2.5_ | | | | | |
| --- | --- | --- | --- | --- | --- |
|  | **High PM_2.5_** number/total | **High PM_2.5_** | | **Log PM_2.5_** | |
|  |  | Odds ratio  (95% CI) | *p* value | Odds ratio  (95% CI) | *p value* |
| **Urban** |  |  |  |  |  |
| Robust | 7290/9233 | 1 (reference) |  | 1 (reference) |  |
| Prefrail | 5950/7320 | 1.4 (1.3–1.5) | **< 0.001** | 4.2 (2.1–8.3) | **< 0.001** |
| Frail | 605/747 | 1.5 (1.2–1.9) | **0.001** | 9.3 (1.6–52.2) | **0.012** |
| **Male** |  |  |  |  |  |
| Robust | 3418/4990 | 1 (reference) |  | 1 (reference) |  |
| Prefrail | 2637/4020 | 2.2 (1.9–2.6) | **< 0.001** | 4.2 (2.2–7.9) | **< 0.001** |
| Frail | 284/486 | 2.1 (1.5–3.1) | **< 0.001** | 1.8 (0.5–6.0) | 0.365 |
| **Female** |  |  |  |  |  |
| Robust | 3872/5394 | 1 (reference) |  | 1 (reference) |  |
| Prefrail | 3313/5122 | 1.0 (0.9–1.1) | 0.992 | 0.8 (0.4–1.6) | 0.588 |
| Frail | 321/594 | 1.2 (0.9–1.6) | 0.257 | 0.7 (0.2–2.2) | 0.498 |
| **Charlson Comorbidity Index < 2** | |  |  |  |  |
| Robust | 7017/10010 | 1 (reference) |  | 1 (reference) |  |
| Prefrail | 5687/8729 | 1.4 (1.2–1.5) | **< 0.001** | 1.8 (1.1–2.8) | **0.013** |
| Frail | 539/980 | 1.4 (1.1–1.7) | **0.016** | 1.1 (0.4–2.5) | 0.910 |
| **Charlson Comorbidity Index ≥ 2** | |  |  |  |  |
| Robust | 273/374 | 1 (reference) |  | 1 (reference) |  |
| Prefrail | 263/413 | 2.1 (1.2–3.8) | **0.012** | 4.6 (0.4–54.6) | 0.232 |
| Frail | 66/100 | 5.1 (2.1–12.6) | **< 0.001** | 2.5 (0.1–86.7) | 0.614 |
| **Age < 75** |  |  |  |  |  |
| Robust | 5436/7614 | 1 (reference) |  | 1 (reference) |  |
| Prefrail | 3640/5396 | 1.3 (1.2–1.5) | **< 0.001** | 2.0 (1.1–3.5) | **0.021** |
| Frail | 199/365 | 1.3 (0.9–1.9) | 0.187 | 0.3 (0.1–1.0) | **0.049** |
| **Age ≥ 75** |  |  |  |  |  |
| Robust | 1854/2770 | 1 (reference) |  | 1 (reference) |  |
| Prefrail | 2310/3746 | 1.5 (1.3–1.8) | **< 0.001** | 2.0 (0.9–4.1) | 0.077 |
| Frail | 406/715 | 1.8 (1.3–2.4) | **< 0.001** | 2.6 (0.8–8.2) | 0.108 |
| **Household income ≥ 19,677 USD**^a^ | |  |  |  |  |
| Robust | 5162/5777 | 1 (reference) |  | 1 (reference) |  |
| Prefrail | 3625/4116 | 0.8 (0.7–1.0) | **0.017** | 2.8 (1.2–6.3) | **0.015** |
| Frail | 387/462 | 0.8 (0.6–1.2) | 0.328 | 12.9 (1.5–112.0) | **0.021** |
| **Household income < 19,677 USD**^a^ | |  |  |  |  |
| Robust | 2128/4607 | 1 (reference) |  | 1 (reference) |  |
| Prefrail | 2325/5026 | 2.7 (2.2–3.3) | **< 0.001** | 2.5 (1.4–4.5) | **0.002** |
| Frail | 218/618 | 4.0 (2.8–5.8) | **< 0.001** | 2.4 (0.8–6.8) | 0.105 |
| **Non-smoker** | |  |  |  |  |
| Robust | 6907/9765 | 1 (reference) |  | 1 (reference) |  |
| Prefrail | 5625/8567 | 1.4 (1.2–1.5) | **< 0.001** | 4.3 (0.8–25.1) | 0.101 |
| Frail | 581/1012 | 1.6 (1.3–2.0) | **< 0.001** | 0.3 (0.0–6.3) | 0.460 |
| **Smoker** |  |  |  |  |  |
| Robust | 383/619 | 1 (reference) |  | 1 (reference) |  |
| Prefrail | 325/575 | 2.5 (1.6–4.0) | **< 0.001** | 4.3 (0.8–25.1) | 0.101 |
| Frail | 24/68 | 0.5 (0.2–1.4) | 0.192 | 0.3 (0.0–6.3) | 0.460 |
| CI, confidence interval; USD, United States Dollars.  ^a^Converted from New Taiwan Dollars (NTD) at a rate of 1 USD = 31 NTD.  Bold type denotes statistical significance. | | | | | |

| **Supplementary Table S 2** Association between frailty status and PM_2.5_ in logistic regression analyses adjusted for chronic diseases. | | | | | |
| --- | --- | --- | --- | --- | --- |
|  | **High PM_2.5_** | **High PM_2.5_** | | **Log PM_2.5_** | |
|  | number/total | Odds ratio  (95% CI)^a^ | *p* value | Odds ratio  (95% CI)^a^ | *p* value |
| **Frailty status** |  |  |  |  |  |
| Robust | 7290/10,384 | 1 |  | 1 |  |
| Prefrail | 5950/9142 | 1.4(1.3-1.5) | **<0.001** | 1.8(1.2-2.6) | **0.003** |
| Frail | 605/1080 | 1.5(1.2-1.9) | **0.001** | 1.1(0.5-2.4) | 0.732 |
| CI, confidence interval.  ^a^Multinomial logistic regression adjusted for age, sex, smoking, hypertension, diabetes, heart disease, stroke and chronic kidney disease and urbanization.  Bold type denotes statistical significance. | | | | | |
